# Various Forms of Hysterical or Functional Paralysis

**Published:** 1893-09

**Authors:** 


*Various Forms of Hysterical or Functional Paralysis.* By H. CHARLTON BASTIAN, M.A., M.D., F.R.S. Pp. xi., 199. London: H. K. LEWIS. 1893.

This book consists of some lectures published in the *Lancet* for 1891, together with subsequent considerable additions, and, in the form of appendices, papers on the "Muscular Sense" and on the "Neural Processes Underlying Attention and Volition," which appeared in *Brain*, and also a short discussion of the question of the double representation of touch and common sensibility in the cerebral hemispheres. Dr. Bastian justly holds that we can only attain to a solution of the vexed question of the nature of hysteria and of functional paralysis by the study in fullest detail of the symptoms presented by patients suffering from these disorders. One feature of the book is therefore the number of fully-reported cases illustrative of the points under discussion. These cases are admirably told, and make very interesting reading. He has also attempted a classification of the various forms of hysterical or functional paralysis, based on the supposed seat of the disturbance of function in the nervous centres. Some systematisation of cases of functional paralysis is urgently needed. The question is, Is our knowledge of them even yet sufficiently wide to enable us to form generalisations? To a certain extent the labours of the many workers in neurology during recent years have resulted in the distinction of several varieties of functional paralysis. A great step in advance would be the use of the term Hysteria by all writers to mean a definite type of nervous disorder characterised by certain easily recognised signs.

Charcot has attempted to do this, and considers hysteria in respect of its origin as a perversion of certain brain functions. The author would still further limit the use of the term, and restrict it to a certain class of functional disorders of his "central" type, and not apply it to all functional motor paralyses of cerebral origin. One feature of this work is the attempt to classify apart cases in which the symptoms correspond with those met with in cerebral paralysis of organic origin and in diseases of the spinal cord, into cases of cerebral and spinal type respectively. A broad distinction of this kind would probably be generally assented to. But here again a difficulty in the use of "hysterical" crops up, for many cases of functional paraplegia present other symptoms, especially on the sensory side, which show them to be closely connected with the more commonly accepted hysterical cases of cerebral type; that is to say, in functional paraplegia we may have to look to the brain rather than to the cord as the primary seat of disturbance.

When we come to the author's subdivision of the two chief types, it seems to us that it may be convenient clinically to subdivide the cases of cerebral types according as the symptoms are analogous to those met with in lesions of the cortex, centrum ovale, and internal capsule; but there is little evidence except from analogy to show that actually the fibres of the internal capsule lose their power of conductivity, and that it is going further than our present knowledge warrants in assigning cases of functional paralysis to functional incapacity of these fibres.

Again, in the cases of spinal type, can instances of spastic functional paralysis be referred to affection of the pyramidal system of fibres? We think that if so it is more reasonable to look for the primary seat of disorder either in their terminal ramifications in the grey matter, or in the trophic cells at their starting point; in short, in all cases of functional paralysis the strong probability is that the seat of the affection is in some part of the grey matter, and not in the white matter, both in the spinal cord and in the brain. Nor do we think that the author has thrown any fresh light on the actual nature of the pathological process that takes place in the nervous centres in functional paralysis. He inclines to the theory of vaso-motor spasm; but there are many difficulties in the way of accepting this. Surely in many cases the changes may be finer than even those which may result from more or less diminished blood supply. As a matter of fact we have little certain knowledge as to the exact nature of the perversion or loss of function which occurs.

To pass from these criticisms, we may say that whatever opinions are held as to the nature and seat of the functional disturbance in hysteria, the author's views must be given full consideration, and will repay careful study, both on account of the ability with which they are urged, and the number of examples he brings forward to sustain them. His wide reading and powers of observation give him a thorough mastery over neurological problems. No one who reads this book carefully can rise from its perusal without feeling that he has gained a wider and broader grasp of the general considerations involved in the study of functional nervous disease, and that he has also added to his store of practical knowledge for his every-day work. The section which explains the process of reasoning and analysis of symptoms which must be gone through in any particular case before the distinctive diagnosis between functional and organic disease can be properly made, is one of great value. We fear that only too often the diagnosis of hysteria is made on the most inadequate grounds, and sometimes with disastrous consequences. That physician who has the widest knowledge of the forms which nervous disease may take will be the most careful in his diagnosis of hysteria. The sections on prognosis and treatment, though short, are of equal merit with the rest of the book.

Space will not allow us to do more than commend to students of psychology the careful consideration of the included papers on Muscular Sense, Attention and Volition. We cannot conclude without strongly recommending this work as a valuable and important contribution to the practical study of functional nervous disease, and at the same time express the hope that it may meet with the success that it deserves.

